# Optimising Large Animal Models of Sustained Atrial Fibrillation: Relevance of the Critical Mass Hypothesis

**DOI:** 10.3389/fphys.2021.690897

**Published:** 2021-06-15

**Authors:** Nathan C. Denham, Charles M. Pearman, George W. P. Madders, Charlotte E. R. Smith, Andrew W. Trafford, Katharine M. Dibb

**Affiliations:** Unit of Cardiac Physiology, Institute of Cardiovascular Sciences, Manchester Academic Health Sciences Centre, The University of Manchester, Manchester, United Kingdom

**Keywords:** atrial, fibrillation, critical, mass, hypothesis, pathophysiology, ovine, model

## Abstract

**Background:**

Large animal models play an important role in our understanding of the pathophysiology of atrial fibrillation (AF). Our aim was to determine whether prospectively collected baseline variables could predict the development of sustained AF in sheep, thereby reducing the number of animals required in future studies. Our hypothesis was that the relationship between atrial dimensions, refractory periods and conduction velocity (otherwise known as the critical mass hypothesis) could be used for the first time to predict the development of sustained AF.

**Methods:**

Healthy adult Welsh mountain sheep underwent a baseline electrophysiology study followed by implantation of a neurostimulator connected via an endocardial pacing lead to the right atrial appendage. The device was programmed to deliver intermittent 50 Hz bursts of 30 s duration over an 8-week period whilst sheep were monitored for AF.

**Results:**

Eighteen sheep completed the protocol, of which 28% developed sustained AF. Logistic regression analysis showed only fibrillation number (calculated using the critical mass hypothesis as the left atrial diameter divided by the product of atrial conduction velocity and effective refractory period) was associated with an increased likelihood of developing sustained AF (Ln Odds Ratio 26.1 [95% confidence intervals 0.2–52.0] *p* = 0.048). A receiver-operator characteristic curve showed this could be used to predict which sheep developed sustained AF (C-statistic 0.82 [95% confidence intervals 0.59–1.04] *p* = 0.04).

**Conclusion:**

The critical mass hypothesis can be used to predict sustained AF in a tachypaced ovine model. These findings can be used to optimise the design of future studies involving large animals.

## Introduction

Atrial fibrillation (AF) is a common arrhythmia characterised by rapid and disorganised atrial electrical activity, resulting in the loss of atrial contractility and an irregular ventricular response (Kirchhof et al., 2016). It is a major burden on healthcare systems worldwide as it is associated with an increased risk of cardiovascular morbidity (Wolf et al., 1991; Stewart et al., 2002) and mortality (Benjamin et al., 1998). A significant part of our current understanding of the underlying pathophysiology can be attributed to the use of large animal models in preclinical studies that aim to replicate the natural history of AF in humans by inducing progressive remodelling of the cardiac atria until a point where the arrhythmia is self-sustaining (Denham et al., 2018). Unfortunately, no model is perfect and it has been recognised that a subset of animals are resistant to developing sustained AF (Willems et al., 2001a). A guiding principle of animal research in the United Kingdom is summarised by the 3Rs (NC3Rs, 2020): replacement (methods to avoid or replace animal use), reduction (methods to minimise the number of animals used in studies) and refinement (methods to minimise suffering). Our aim was to prospectively collect baseline physiological variables known to influence the vulnerability to AF in humans and determine which (if any) improved the ability to predict the development of sustained AF in our ovine tachypacing model. This knowledge could then be applied to improve future experimental efficiency by reducing the overall number of animals required to produce sustained AF.

The current understanding of the pathophysiology of AF has been extensively reviewed elsewhere (Heijman et al., 2014; Denham et al., 2018). A key tenet is the critical mass hypothesis which states that: the probability of sustaining AF is proportional to the dimensions of the atrial tissue divided by the wavelength (the atrial effective refractory period times the conduction velocity) (Moe et al., 1964). In brief, AF is believed to be formed by multiple re-entrant wavelets of excitation whose optimal size is determined by the wavelength. The shorter the wavelength (generated by atria with a shorter effective refractory period and slower conduction velocity), the more wavelets can be sustained at the same time in a fixed volume of tissue reducing the likelihood of all extinguishing concurrently terminating AF (Moe et al., 1964). A larger atrial tissue dimension on the other hand will allow for more wavelets of a fixed size to be accommodated at the same time, reducing the likelihood all will terminate at a single time point. Our original hypothesis was that in a cohort of healthy sheep devoid of any pre-existing pathological remodelling, animals that could sustain the greatest number of re-entrant wavelets according to the critical mass hypothesis would be more likely to develop sustained AF during a tachypacing protocol.

## Methods

All procedures were in accord with The UK Animals (Scientific Procedures) Act (1986) and EU directive 2010/63. Institutional approval was received from The University of Manchester Animal Welfare and Ethical Review Board. Reporting follows the ARRIVE (Animal Research: Reporting of *in vivo* Experiments) guidelines (Kilkenny et al., 2010).

### Animal Model

A total of 19 adult female Welsh mountain sheep of approximately 18 months of age were used in this study. Only female sheep were included due to the limited availability of aged-matched males as a result of standard United Kingdom farming practices performing castration in the postnatal period. Sheep were group housed in a facility maintained on a 12-h light/12-h dark cycle, and afforded amble bedding and a diet of hay. All were free of observable disease and had acclimatised to the housing facility for a minimum of 1 week prior to starting the study. Data were included for all sheep that completed the pre-defined experimental protocol. One sheep was excluded from analysis who failed to complete the protocol due to displacement of the atrial pacing lead into the ventricle, requiring euthanasia as a humane end-point.

### Atrial Fibrillation Protocol

Sheep were anaesthetised with 3% v/v isoflurane and right jugular venous access was performed via cut down and venotomy. Permanent pacing leads were advanced to the right ventricular apex and the right atrial appendage, after which they were connected to a dual chamber internal cardiac defibrillator (Medtronic, Woburn, MA). The defibrillator allowed for the wireless recording of atrial and ventricular electrograms in the experimental sheep, minimising any confounding effects of the stress associated with frequent handling on the inducibility of atrial fibrillation. A third pacing lead was placed in a separate location in the right atrial appendage and connected to a Medtronic Itrel-4 neurostimulator. The neurostimulator was programmed to deliver intermittent 50 Hz burst pacing in a repetitive cycle of 30 s on, 30 s off; at a minimum output of three times pacing threshold (range 3–5 V) with a pulse width of 390 ms. The 30 s on/off cycle was chosen to allow for the quantification of AF duration during the off period on a daily basis, minimising the handling that would have been required to deactivate the neurostimulator if it had been programmed for continuous pacing. All leads were secured to the adjacent muscle with 2’0 silk sutures and the device generators were buried in separate subcutaneous pockets. The wound was closed in two layers with 2’0 Vicryl sutures and the sheep were recovered. Peri-operative analgesia and antimicrobials were given in the form of subcutaneous meloxicam (1 mg/kg bodyweight; Boehringer Ingelheim, Ingelheim am Rheim, Germany) and intramuscular Betamox (15 mg/kg bodyweight; Norbrook Laboratories, Newry, United Kingdom), respectively.

After a minimum of 1 week recovery period, the neurostimulator was activated for a duration of 8 weeks. The 8-week duration of the study was chosen based on unpublished pilot data from our group (*n* = 4), performed to establish the timeframe an intermittent pacing protocol would produce sustained AF in sheep. The daily burden of AF was monitored via wireless communication with the defibrillator, measuring the duration of AF during the 30 s off period. At the end of the 8-week protocol, the presence of sustained AF was documented, defined by deactivating the neurostimulator in the conscious sheep and recording 15 min of uninterrupted AF. At the end of the 8-week period, sheep were euthanised by permanent interruption of the circulation under terminal anaesthesia with pentobarbitone (200 mg/kg bodyweight; AnimalCare, York, United Kingdom).

### Pre-operative Assessment

Prior to surgery, bodyweight was recorded and the sheep in a casted position underwent a conscious transthoracic echocardiogram using a Vivid 7 ultrasound system (General Electric, Boston, MA). Left atrial anteroposterior diameter was measured in the parasternal long axis view as the mean over three cardiac cycles, as apical views could not be obtained in the sheep to assess left atrial volume. Epicardial fat pad thickness was measured anterior to the right ventricle in the parasternal long axis view using the atrioventricular groove as an anatomical reference, as per the method described by Iacobellis et al. (2003). An example echocardiographic image of measurements is provided in Supplementary Figure 1.

### Electrophysiology Studies

A 5-lead electrocardiogram was recorded under general anaesthesia using Emka (Emka Technologies, Paris, France) and analysed in LabChart version 7 (AD instruments, Colorado Springs, CO). *P* wave duration was taken as the maximum measurement recorded in any lead, and along with PR interval were averaged over 10 cycles in sinus rhythm prior to incision. Monophasic action potentials (MAPs) were recorded from the posterior wall of the right atrium using a Blazer electrophysiology catheter (Boston Scientific, Quincy, MA). Signals were digitised at 1 kHz using a PowerLab amplifier and LabChart version 7. The atrial effective refractory period (AERP) was determined by programmed electrical stimulation through the implanted defibrillator using a basic drive train of eight beats at 400 ms cycle length, followed by an extrastimulus starting at a coupling interval of 350 ms and decrementing in 10 ms intervals until the AERP was reached. The AERP was evidenced by the longest coupling interval which failed to produce a MAP, recorded using the Blazer electrophysiology catheter. The rate threshold for atrial MAP alternans was determined by delivering fixed burst atrial stimulation through the defibrillator over the cycle length range 500–250 ms. Alternans was assessed using 32-beat trains of action potentials and analysed using a spectral method as described previously (Pearman, 2014; Pearman et al., 2018), defining MAP alternans as trains with both a k-score ≥ 3 and a V_Alt_ ≥ 0.02 mV. The rate threshold was the longest paced cycle length needed to produce atrial MAP alternans. The mean duration of induced AF was calculated after 10 bursts of 50 Hz noise at an output of 5 V for 5 s. Atrial conduction velocity was recorded using a 2/8/2 decapolar electrophysiology catheter (Biosense Webster, Irvine, CA) placed on the lateral wall of the right atrium. Conduction velocity was calculated as the time taken to register a bipolar electrogram from the distal bipoles whilst pacing from the proximal bipoles via a Medtronic 2292 surgical pacing cable (Medtronic, Woburn, MA) attached to a Medtronic Carelink 2090 programmer. Right atrial conduction velocity was measured at the same cycle length as the AERP (400 ms) as well as the maximum rate available through the stimulator (cycle length 273 ms) in order to better approximate the rapid atrial electrical activity expected in AF.

### Data Analysis and Statistics

Atrial wavelength was calculated using Equation 1, utilising conduction velocity measured at a paced cycle length of 400 and 273 ms. The relationship of left atrial size to wavelength (hereto described as fibrillation number) was calculated using Equation 2 which was adapted from Hwang et al. (2015).

(1) $$Wavelength\left(cm\right)=AERP\left(ms\right)XAtrialconduction\;velocity\left(cm\cdot ms^{-1}\right)$$

(2) $$Fibrillationnumber=\frac{Leftatrialanteroposteriordiameter\left(cm\right)}{Wavelength\left(cm\right)}$$

Raw data are reported as mean ± standard error of the mean (SEM) and logistic regression data as natural logarithms of odds ratio (Ln OR) with the 95% confidence intervals (95%CI). Statistical testing and presentation of continuous variables, logistic regression and receiver-operator characteristic (ROC) curve analysis was performed using Sigmaplot V11 (Systat Software, San Jose, CA). A two-tailed Student’s *t*-test was used to compare continuous data where differences were considered significant when *p* < 0.05. Logistic regression confidence intervals which crossed zero where considered non-significant. ROC curve analysis was considered non-significant where the 95%CI of the C-statistic crossed 0.5. Optimal cut-off valves were determined from ROC curves with significant predictive value by calculating the Youden J statistic (Equation 3), where sensitivity and specificity are expressed numerically between zero and one. The Youden J statistic is a dimensionless index between zero and one, where a score of zero means the independent variable is no better a predictor than random chance, whereas a score of one represents a perfect predictor of sustained AF.

(3) YoudenJstatistic=(Sensitivity+Specificity)-1

## Results

Eighteen sheep completed the 8-week pacing protocol, of which 5/18 (28%) developed sustained AF. The remaining 13/18 (72%) sheep were unable to sustain 15 min of uninterrupted AF and were classified as resistant. All 18 sheep underwent the baseline *in vivo* assessment of pre-specified variables, however, the rate threshold for atrial MAP alternans could only be determined in 15/18 (83%).

### Sheep Who Developed Sustained Atrial Fibrillation Have a Larger Fibrillation Number at Baseline

An overview of the baseline characteristics between the sustained AF and resistant cohort can be observed in Table 1. Variability in the characteristics between sheep in the absence of pre-existing disease reflects genetic variation in an outbred population. No significant differences between the groups were seen when looking at single variables alone, although the AERP trended toward being shorter in those who developed sustained AF (132 ± 7 ms vs. 155 ± 6 ms; *p* = 0.05). When variables were combined according to the critical mass hypothesis to produce wavelength and fibrillation number, no difference was observed at a paced cycle length of 400 ms (wavelength: 12.0 ± 0.85 cm vs. 12.6 ± 0.54 cm, *p* = 0.59; fibrillation number: 0.22 ± 0.02 vs. 0.20 ± 0.01, *p* = 0.32). In contrast, when wavelength and fibrillation number were recalculated at the shortest paced cycle length of 273 ms, both a shorter wavelength (9.9 ± 0.8 cm vs. 12.7 ± 0.7 cm; *p* = 0.04) and a larger fibrillation number (0.27 ± 0.03 vs. 0.20 ± 0.01; *p* = 0.02) was observed in the group developing sustained AF. According to the critical mass hypothesis, this suggests naive sheep who go on to develop sustained AF are capable of sustaining a greater number of re-entrant fibrillatory wavelets for a fixed volume of atrial tissue, lowering the probability of AF terminating at a single point in time. As the AF cohort was small (*n* = 5), it was not possible to determine which variable (shorter AERP, slower conduction velocity or larger left atrial diameter at baseline) had the greatest influence on the larger fibrillation number.

**TABLE 1:** Differences in baseline characteristics between the sheep developing sustained atrial fibrillation (*N* = 5) and those who were resistant (*N* = 13).

Single variables (units)	Sustained AF	Resistant	Significance
*P* wave duration (ms)	50.6 ± 2.8	45.7 ± 2.9	*P* = 0.33
PR interval (ms)	109.6 ± 6.3	109.6 ± 2.4	*P* = 0.99
AERP (ms)	132 ± 7	155 ± 6	*P* = 0.05
Mean duration of induced AF (s)	35.2 ± 29.0	6.4 ± 3.4	*P* = 0.13
Weight (kg)	37.8 ± 2.1	36.2 ± 1.3	*P* = 0.53
Left atrial diameter (cm)	2.62 ± 0.10	2.46 ± 0.05	*P* = 0.11
Epicardial fat pad thickness (cm)	0.70 ± 0.11	0.63 ± 0.07	*P* = 0.60
Conduction velocity (CL400 ms) (m/s)	0.93 ± 0.09	0.82 ± 0.04	*P* = 0.27
Conduction velocity (CL273 ms) (m/s)	0.76 ± 0.08	0.83 ± 0.05	*P* = 0.51
Rate threshold atrial alternans (ms)	308 ± 9	313 ± 8	*P* = 0.71
**Combined variables**			
Wavelength (CL 400 ms) (cm)	12.0 ± 0.85	12.6 ± 0.54	*P* = 0.59
**Wavelength** (**CL 273 ms**) **(cm)**	**9.9 ± 0.8**	**12.7 ± 0.7**	***P* = 0.04**
Fibrillation number (CL400 ms)	0.22 ± 0.02	0.20 ± 0.01	*P* = 0.32
**Fibrillation number** (**CL273 ms**)	**0.27 ± 0.03**	**0.20 ± 0.01**	***P* = 0.02**

*Data presented as mean ± SEM; statistical comparisons using an unpaired t-test; statistical significance taken as p < 0.05. AERP, atrial effective refractory period; AF, atrial fibrillation; EFT, epicardial fat pad thickness; CL, paced cycle length.*

### A Larger Fibrillation Number Increases the Probability of Developing Sustained AF

We next asked whether any of the aforementioned variables could increase the probability of developing AF. A logistic regression analysis showed none of the single baseline variables were associated with an increased likelihood of developing sustained AF (Table 2). When the combined variables of wavelength and fibrillation number were analysed, wavelength was not associated with an increased likelihood of developing sustained AF at either a paced cycle length of 400 ms (Ln OR −17.0 [95%CI −74.4 to 40.4]) or 273 ms (Ln OR −64.2 [95%CI −134.7 to 6.4]). In contrast, fibrillation number was associated with an increased likelihood of developing sustained AF at a paced cycle length of 273 ms (Ln OR 26.1 [95%CI 0.2–52.0] *p* = 0.048) but not at a paced cycle length of 400 ms (Ln OR 13.8 [95%CI −12.6 to 40.2]). In the context of the critical mass hypothesis, this means the larger the fibrillation number at baseline, the greater the likelihood of developing sustained AF.

**TABLE 2:** Univariate logistic regression analysis of single baseline characteristics between the sheep who develop sustained AF and those who were resistant.

Variable (units)	Ln OR	95% CI
*P* wave duration (ms)	0.06	−0.05 to 0.18
PR interval (ms)	0	−0.11 to 0.11
AERP (ms)	−0.05	−0.11 to 0.01
Mean duration of induced AF (s)	0.03	−0.02 to 0.07
Weight (kg)	0.09	−0.17 to 0.34
Left atrial diameter (cm)	6.25	−1.74 to 14.24
Epicardial fat pad thickness (cm)	1.15	−2.94 to 5.25
Conduction velocity (CL400 ms) (m/s)	3.56	−2.71 to 9.84
Conduction velocity (CL273 ms) (m/s)	−2.10	−8.07 to 3.87
Rate threshold atrial alternans (ms)	−0.01	−0.06 to 0.04
Wavelength (CL 400 ms) (cm)	−17.0	−74.43 to 40.40
Wavelength (CL 273 ms) (cm)	−64.18	−134.74 to 6.39
Fibrillation number (CL400 ms)	13.83	−12.59 to 40.25
Fibrillation number (CL273 ms)	26.12	0.22 to 52.01

*Data presented as natural logarithms of odds ratio (Ln OR) with 95% confidence intervals (CI). 95% CI which cross zero are non-significant. AERP, atrial effective refractory period; CL, paced cycle length.*

### Fibrillation Number Can Be Used to Predict the Development of Sustained AF

**FIGURE 1:**
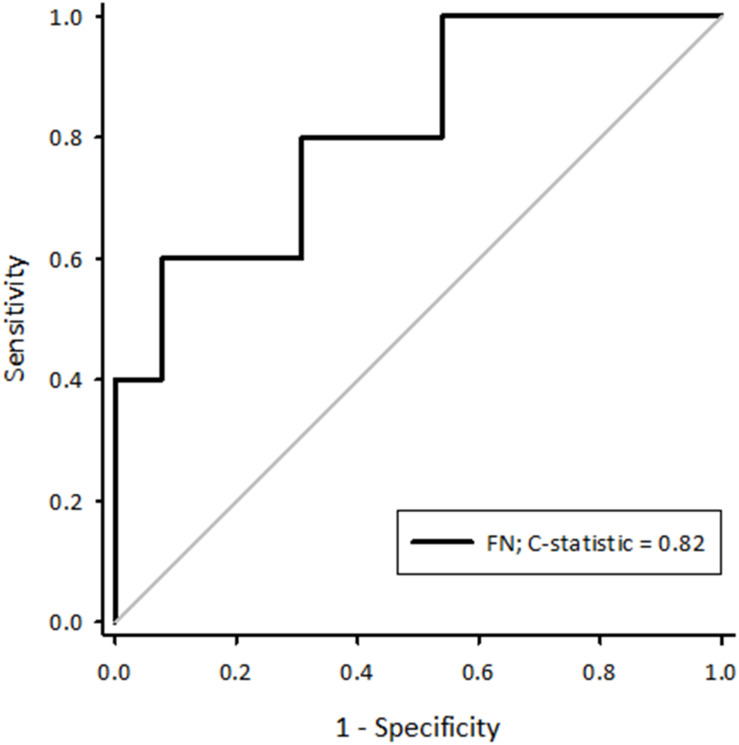
A receiver-operator characteristic curve generated for fibrillation number calculated using a paced cycle length of 273 ms. The C-statistic was 0.82 (95%CI 0.59–1.04, *p* = 0.04). FN, fibrillation number.

A receiver-operator characteristic (ROC) curve was created for fibrillation number calculated using a paced cycle length of 273 ms (Figure 1). This generated a C-statistic of 0.82 (95%CI 0.59–1.04, *p* = 0.04) demonstrating it is therefore possible to predict the likelihood of a sheep developing sustained AF using baseline variables prior to any pathological remodelling of the atria. To maximise the predictive power of the curve, the optimal cut-offs were calculated. The highest Youden J statistic was attributed to a fibrillation number > 0.28 (sensitivity 60%, specificity 92%, *J* = 0.52). In summary, this suggests sustained AF had a greater likelihood of being observed in sheep with a combination of a larger left atrial diameter, a slower atrial conduction velocity measured at a paced cycle length of 273 ms and a shorter AERP. As such, although these parameters alone did not influence the likelihood of sustained AF, they could collectively be positively selected for to minimise the number of experimental animals who are resistant to developing sustained AF.

## Discussion

### Relevance of the Critical Mass Hypothesis to Developing Sustained AF in Tachypaced Sheep

In this study, only fibrillation number was shown to be suitably predictive of sustained AF in sheep who underwent our 8-week tachypacing protocol. None of the constituent variables (atrial diameter, conduction velocity and effective refractory period) or the wavelength were significantly useful alone in predicting sustained AF. This supports our original hypothesis that it is the combination of the three variables which underpins the critical mass hypothesis and best predicts the likely experimental outcome. The critical mass hypothesis has previously been used to predict the duration of AF induced acutely in animals over a short timeframe of pacing [minute to hours (Byrd et al., 2005; Lee et al., 2013)] and also in humans with longstanding AF with significant atrial remodelling to predict treatment success (Hwang et al., 2015; Williams et al., 2019). As such, this is the first study to use the critical mass hypothesis to aid the prediction of sustained AF in long term animal models who also have no known pre-existing pathological atrial remodelling. This finding would support the critical mass hypothesis as part of the key mechanism underpinning the pathophysiology of AF, which currently remains incompletely understood particularly what the basic re-entrant wavelet in AF represents (Nattel et al., 2017).

### Fibrillation Number Can Be Used to Improve Experimental Efficiency in Our Model

Only 28% of sheep developed sustained AF during the experimental protocol. In line with the 3Rs of animal experimentation (NC3Rs, 2020), there is clearly room to improve experimental efficiency by reducing the number of resistant animals who ultimately go through the surgery and experimental protocol without reaching the desired endpoint. Our study has shown fibrillation number can be used to assist in predicting which animals are likely to develop sustained AF, however, it should be noted fibrillation number can only be calculated after an electrophysiology study. As such, sheep would need to undergo an invasive procedure prior to calculating their likelihood of developing sustained AF. In this situation, it is suggested animals with a low probability of developing sustained AF could be triaged to alternative experimental studies rather than embark upon an 8-week protocol which has a low likelihood of creating the desired endpoint (NC3Rs, 2020).

### Wider Implications for Ovine Models of Atrial Fibrillation

A number of experimental AF protocols have been described in the literature, the commonest based upon rapid atrial pacing (Willems et al., 2001a,b; Anne et al., 2007; Pruvot et al., 2007; Lenaerts et al., 2009, 2013; Filgueiras-Rama et al., 2012; Martins et al., 2014; Monigatti-Tenkorang et al., 2014; Macquaide et al., 2015; Takemoto et al., 2016, 2017; Haemers et al., 2017) although methods involving autonomic modulation (Geddes et al., 1996) or structural insults (Deroubaix et al., 2004) have been described. At present, there is no protocol which is considered the gold standard for producing sustained AF in sheep. A summary of the reported methodologies relevant to tachypaced ovine models of sustained AF is produced in Table 3.

**TABLE 3:** A summary of studies in the literature utilising a tachypacing methodology to induce sustained atrial fibrillation in sheep.

Tachypaced AF protocol: FIRST author and references	Methodology of tachypacing	Defintion of sustained AF	Success rate (% sheep developing sustained AF by the end of the protocol)
Willems et al. (2001a,b), Anne et al. (2007)	Continuous right atrial pacing at cycle length 67 ms for a mean of ∼6 months	AF lasting > 1 h after cessation of pacing	70–77%
Pruvot et al. (2007), Monigatti-Tenkorang et al. (2014)	Intermittent right atrial pacing at cycle length 170 ms for 8 weeks. Stepwise protocol starting 2 s on/5 s offuntil maximum 5 s on/2 s off	AF lasting > 1 week after cessation of pacing	100%
Lenaerts et al. (2009, 2013), Macquaide et al. (2015), Haemers et al. (2017)	Continuous right atrial pacing at cycle length 100 ms for 15 weeks	Absence of sinus rhythm after cessation of pacing for the remaining follow up period until euthanasia	100%
Filgueiras-Rama et al. (2012), Martins et al. (2014), Takemoto et al. (2016, 2017)	Intermittent atrial pacing at cycle length 50 ms for 30 s duration followed by 10 s sensing for a range of 9–24 weeks	AF lasting > 1 week after cessation of pacing	100%

*AF, atrial fibrillation.*

Key differences between tachypaced protocols can be categorised by: the nature of pacing (the stimulation frequency and whether it is delivered continuously or intermittently); the site of stimulation (right or left atrium); and the duration of pacing (range: 8 weeks to 6 months). These have been reported to have much better success rates (between 29 and 100%) than seen in our study (Willems et al., 2001a,b; Anne et al., 2007; Pruvot et al., 2007; Lenaerts et al., 2009, 2013; Filgueiras-Rama et al., 2012; Martins et al., 2014; Monigatti-Tenkorang et al., 2014; Macquaide et al., 2015; Takemoto et al., 2016, 2017; Haemers et al., 2017), however, there is no way of accounting for potential underreporting of alternative protocols with higher failure rates. None of these reported protocols exactly matches our own, which we designed specifically to remotely monitor the daily inducibility of AF after intermittent pacing to study the influences on progressive atrial remodelling. Only a single study by Willems et al. (2001a) has directly compared two different tachypacing protocols between one based on continuous stimulation (right atrial pacing at cycle length 100 ms) vs. one based on intermittent stimulation (2.5 s right atrial pacing at cycle length 24 ms, whenever sinus rhythm was detected). Although neither methodology exactly matched our own, continuous tachypacing was more successful in producing sustained AF after 15 weeks (77 vs. 29%). It is therefore possible our low success rate, at least in part, reflects our chosen methodology and either continuous pacing or longer period of intermittent tachypacing may have influenced the number of sheep who went on to develop sustained AF. Whether the use of fibrillation number will show similar predictive power to significantly improve experimental efficiency in other ovine AF models is presently unclear, however, this work highlights the need to critically review and optimise animal models of human pathology wherever possible.

### Limitations

There are a number of limitations which should be considered with regard to the applicability of these results. Firstly, the methodology used in calculating fibrillation number (Equation 2) relied upon a number of assumptions. The dimensions of the atrial tissue were measured using the left atrial anteroposterior diameter calculated on echocardiography, however, it is known this can be a poor estimator of atrial tissue volume (Lester et al., 1999) which is central to the critical mass hypothesis (Byrd et al., 2005; Lee et al., 2013). Unfortunately given limited ultrasound windows available in the majority of sheep, left atrial surface area or volume could be accurately calculated. We therefore used left atrial diameter as it was easily reproducible and has been used in other studies (Hwang et al., 2015). A second limiting factor in calculating fibrillation number was that the wavelength was calculated by performing an electrophysiology study in the right atrium. The left atrium has more relevance to AF seen in humans, particularly as it is the common site where AF is initiated secondary to rapid firing emanating from the pulmonary veins (Haissaguerre et al., 1998). Small differences in the effective refractory period and conduction velocities between the right and left atria are recognised, where the right atrium can only act as a surrogate marker for left atrial electrophysiology (Li et al., 2001; Prabhu et al., 2017). Unfortunately, we were unable to perform a left atrial study due to access issues across the interatrial septum or via a retrograde aortic approach, therefore small differences in fibrillation number may have arisen using right atrial parameters which alter the predictive power for sustained AF.

A further limitation relates to how we defined sustained atrial fibrillation. We believe our requirement for 15 min of uninterrupted AF reflects that the sheep have undergone a significant amount of atrial remodelling, such that they show similar pathophysiology to humans with persistent AF [defined as continuous AF lasting longer than 7 days (Hindricks et al., 2020)]. Other groups have typically defined sustained AF by a longer duration of continuous arrhythmia in their large animal models (Willems et al., 2001a; Lenaerts et al., 2009), therefore it is unlikely the low success rate of our protocol is due to an abnormally high threshold for defining sustained AF.

## Conclusion

In conclusion, we have shown for the first time that we can predict the likelihood of healthy sheep developing sustained atrial fibrillation after the initiation of an 8-week tachypacing protocol through utilising the critical mass hypothesis. Future research studies involving longstanding AF should consider how the relationship between the dimensions of the atrial tissue and the wavelength can help determine the likelihood of animals reaching arrhythmia endpoints. This has the potential to improve future experimental efficiency by reducing the number of animals required and minimising distress in animals resistant to developing sustained AF.

## Data Availability Statement

The raw data supporting the conclusions of this article will be made available by the authors, without undue reservation.

## Ethics Statement

The animal study was reviewed and approved by the University of Manchester Animal Welfare and Ethical Review Board.

## Author Contributions

ND, AT, and KD had the idea for and designed the study. ND, CP, GM, and CS conducted the experiments. ND analysed the data and wrote the manuscript. All authors critically revised the final manuscript and approve of its content.

## Conflict of Interest

The authors declare that the research was conducted in the absence of any commercial or financial relationships that could be construed as a potential conflict of interest.

## Supplementary Material

Supplementary Figure 1 A typical parasternal long axis (PLAX) view obtained by 2D echocardiography from a sheep. Left atrial (LA) anteroposterior diameter was measured during ventricular systole. Epicardial fat pad thickness (EFT) was measured anterior to the right ventricle using the atrioventricular groove as a reference.
