# Supplementary figures and images for: Optimising Large Animal Models of Sustained Atrial Fibrillation: Relevance of the Critical Mass Hypothesis
Source: Front Physiol. 2021 Jun 15;12:690897. doi: 10.3389/fphys.2021.690897 (PMC8239221; doi:10.3389/fphys.2021.690897)

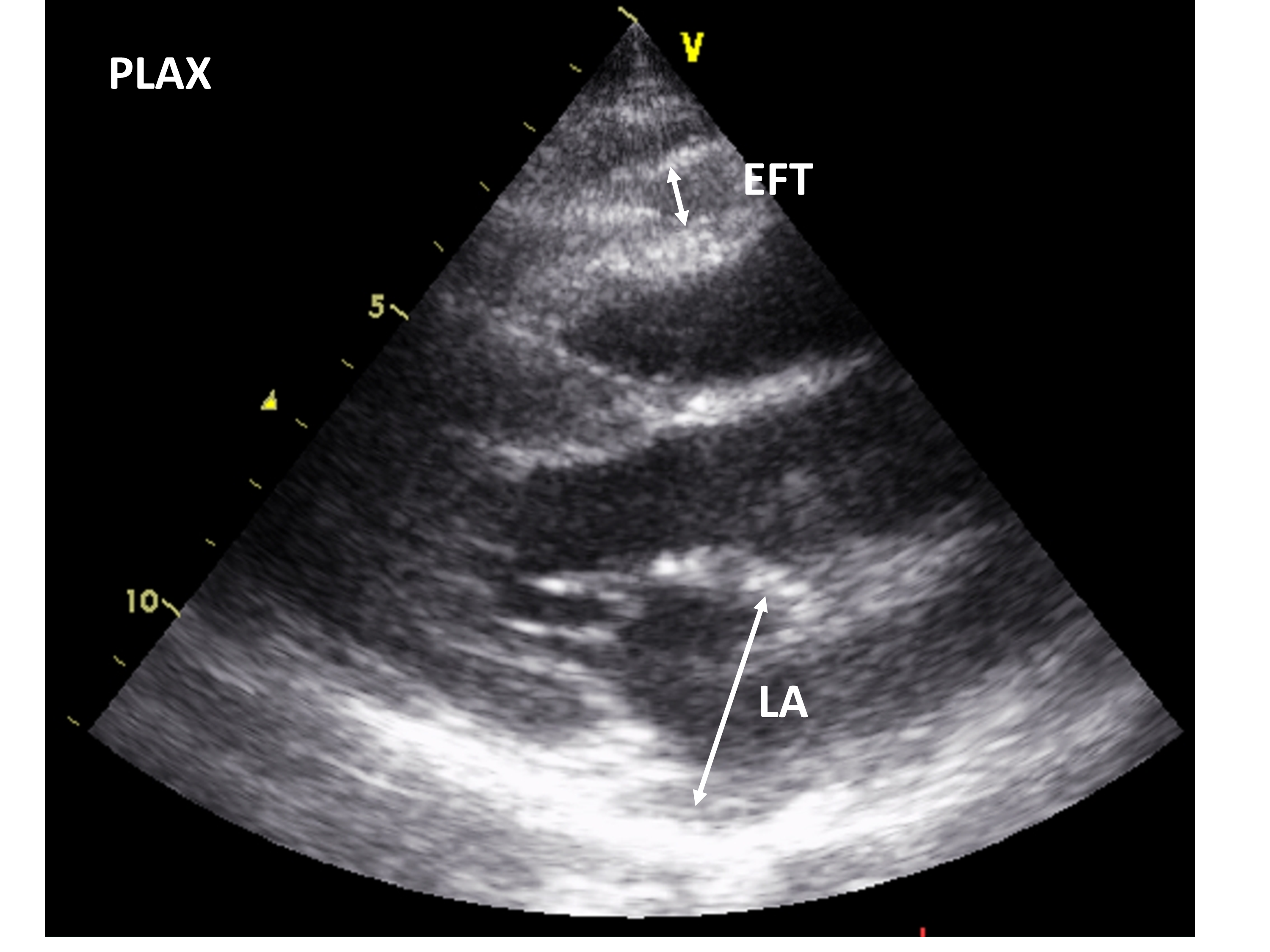

Supplement: Supplementary Figure 1 — A typical parasternal long axis (PLAX) view obtained by 2D echocardiography from a sheep. Left atrial (LA) anteroposterior diameter was measured during ventricular systole. Epicardial fat pad thickness (EFT) was measured anterior to the right ventricle using the atrioventricular groove as a reference. [file Image_1.TIF]
